# Sex Differences in Categorical Adaptation for Faces and Chinese Characters during Early Perceptual Processing

**DOI:** 10.3389/fnhum.2017.00656

**Published:** 2018-01-12

**Authors:** Cuiyin Zhu, Xiaoli Ma, Lihong Ji, Shuang Chen, Xiaohua Cao

**Affiliations:** ^1^Department of Psychology, Zhejiang Normal University, Jinhua, China; ^2^Hangzhou Seventh People's Hospital, Hangzhou, China

**Keywords:** sex, N170, categorical adaptation, face, Chinese character

## Abstract

Previous event-related potential studies support sex differences in the N170 response during face and word processing; however, it remains unclear whether N170 categorical adaptation for faces and words is different between women and men. Using an adaptation paradigm, in which an adaptor and subsequent test stimulus are presented during each trial, the present study investigated N170 categorical adaptation for faces and Chinese characters in both women and men. The results demonstrated that the N170 amplitude elicited by test stimuli in within-category condition was lower than in control category condition, and this was observed during both face and Chinese character processing in women and men. In addition, we found that men have greater N170 categorical adaptation for face and word processing than women. There was also a significant correlation between N170 categorical adaptation indices for face and Chinese character processing in men, which did not occur in women. These findings suggest that men and women process repeated faces or words differently.

## Introduction

Adult humans are proficient at recognizing both human faces and words. Faces and words are objects of expertise. Researchers have identified several behavioral features of expertise in perceptual processing, such as in holistic processing (Richler and Gauthier, 2014); neural features have also been identified, such as specific brain processing areas including the fusiform face area (Kanwisher et al., 1997) and the visual word form area (Cohen and Dehaene, 2004). Previous studies of event-related potentials (ERPs) support the hypothesis that there are specific N170 responses during facial and word perception. For example, the N170 response evoked by faces is often greater than that evoked by non-face objects (Bentin et al., 1996; Eimer, 2000a; Itier and Taylor, 2004; Rossion and Jacques, 2008) and the N170 response to words is much stronger than for letter strings (Bentin et al., 1999; Maurer et al., 2005b) or line drawings (Cao and Zhang, 2011; Cao et al., 2011; Lin et al., 2011; Yum et al., 2011; Zhao et al., 2012). Furthermore, recent ERP studies have demonstrated an important neural trait, the N170 categorical adaptation effect, by measuring the N170 response to face and word processing (Eimer et al., 2010, 2011; Cao et al., 2014b, 2015b).

Categorical adaptation, which is sometimes described as repetition suppression, refers to a category-specific reduction in neuronal activity in response to a repeated stimulus compared to an unrepeated stimulus, although there are some differences between repetition suppression and categorical adaptation (Grill-Spector et al., 2006). In a commonly used adaptation paradigm, an adaptor and subsequent test stimulus are presented during each trial. In the N170 adaptation paradigm, the adaptor and test stimuli are presented successively, with a specified inter-stimulus interval (ISI). The N170 categorical adaptation effect is measured by comparing N170 amplitudes elicited by the same category of test stimulus when preceded by different categories of adaptor stimuli. The N170 adaptation paradigm has been used to characterize neural responses to objects of expertise. For example, many studies have demonstrated that N170 amplitudes are attenuated during face tests that are preceded by face adaptors rather than by non-face adaptors (e.g., houses), suggesting a face-related categorical adaptation effect when there is a short ISI (e.g., 200 ms) (Nemrodov and Itier, 2011; Cao et al., 2014b). Similar to the face-related N170 categorical adaptation effect, word-related effects have been demonstrated using short ISIs (e.g., 200 ms) (Cao et al., 2014a,b, 2015b). Previous studies have also demonstrated that objects of non-expertise cannot produce N170 categorical adaptation similar to that produced by objects of expertise (Cao et al., 2015a). Together, the findings suggest that N170 categorical adaptation is a common phenomenon during visual processing of various expert stimuli presented with short ISIs.

Sex differences in the neural response to objects of expertise have been investigated during early perception (Proverbio et al., 2006, 2010, 2012; Sun et al., 2010). For example, during early perceptual processing, research has consistently demonstrated a right-lateralized face-related N170 response in men, and a bilateral response in women (Proverbio et al., 2006, 2010, 2011, 2012; Godard and Fiori, 2010; Godard et al., 2013; Ji et al., 2016). Only a few studies have used ERPs to investigate sex differences in word processing, although they have found evidence for differences between women and men (Skrandies et al., 1999; Hill et al., 2006; Ji et al., 2016; however, Wirth et al., 2007). However, most previous studies have used ERPs to investigate sex differences in N170 responses to faces or words separately. A recent ERP study examined sex differences in the N170 response evoked by both faces and words (Ji et al., 2016), and found that the N170 response to faces in men was right-lateralized, whereas it was bilateral in women. Moreover, the N170 response to Chinese characters was bilateral in men, whereas it was left-lateralized in women.

The studies described above demonstrate that N170 categorical adaptation is consistent for facial and word processing when a short ISI occurs. Moreover, previous studies have demonstrated sex differences in N170 amplitudes for faces and words (Skrandies et al., 1999; Hill et al., 2006; Proverbio et al., 2006, 2010, 2012; Ji et al., 2016). However, sex differences in N170 categorical adaptation for both face and word processing have not been elucidated. Although previous studies have demonstrated relationships between face and word processing (Nestor et al., 2013; Dehaene et al., 2015), it remains unclear whether there is a correlation between face-related N170 adaptation and word-related N170 adaptation. Therefore, we examined sex differences in N170 categorical adaptation for both face and word processing, in adult participants. We chose objects of long-term expertise (faces and Chinese characters) as the stimuli. Given the existing ERP evidence for sex differences in face (Proverbio et al., 2006, 2010, 2011, 2012; Godard and Fiori, 2010; Godard et al., 2013) and word processing (Skrandies et al., 1999; Hill et al., 2006), we predicted sex differences in N170 categorical adaptation for both face and Chinese character processing. In addition, since many previous studies have demonstrated relationships between face and word stimuli (Nestor et al., 2013; Dehaene et al., 2015), we predicted that the degree of N170 categorical adaptation for faces would be similar to the degree of N170 categorical adaptation for Chinese characters.

## Methods

### Participants

Seventy native Chinese participants (34 women; total sample age range, 19–28 years and mean age, 23.4 years) were recruited from Zhejiang Normal University and received remuneration for their participation. All participants had normal or corrected-to-normal vision, and were right-handed. All participants provided written informed consent, and the protocol was approved by the ethical committee of Zhejiang Normal University.

### Stimuli

The stimuli were grayscale pictures of faces, houses, and Chinese characters. Faces were images of 72 individuals (36 men and 36 women), selected from a standard set of faces at our laboratory; the images displayed neutral facial expressions and were modified to eliminate external features (hair, ears, and jaw line). Adobe Photoshop CS5 was used to homogenize facial contours to a single oval shape, similarly to the process used by Eimer et al. (2010). Seventy-two high frequency Chinese characters with a left-right configuration and 7–14 strokes were chosen from the Modern Chinese frequency dictionary (1986), and were presented in Song font. Seventy-two grayscale images of houses without non-house related objects (e.g., trees) were used as adaptor control stimuli. We used Adobe Photoshop CS5 to eliminate non-house related objects from the house pictures. The face stimuli were 180 × 276 pixels, subtending an angle of 4.0 × 6.2° from a viewing distance of 90 cm. The Chinese character and house stimuli were 198 × 198 pixels in size, subtending an angle of 4.5 × 4.5° from a viewing distance of 90 cm.

### Procedure

Participants sat in a dimly lit room on a chair, 90 cm away from a 17-inch cathode ray tube monitor (1,024 × 768-pixel resolution), on which all stimuli were presented against a dark gray background. E-Prime 2.0 was used for stimulus presentation and behavioral response collection (Psychology Software Tools, Pittsburgh, PA, USA).

**Figure 1:**
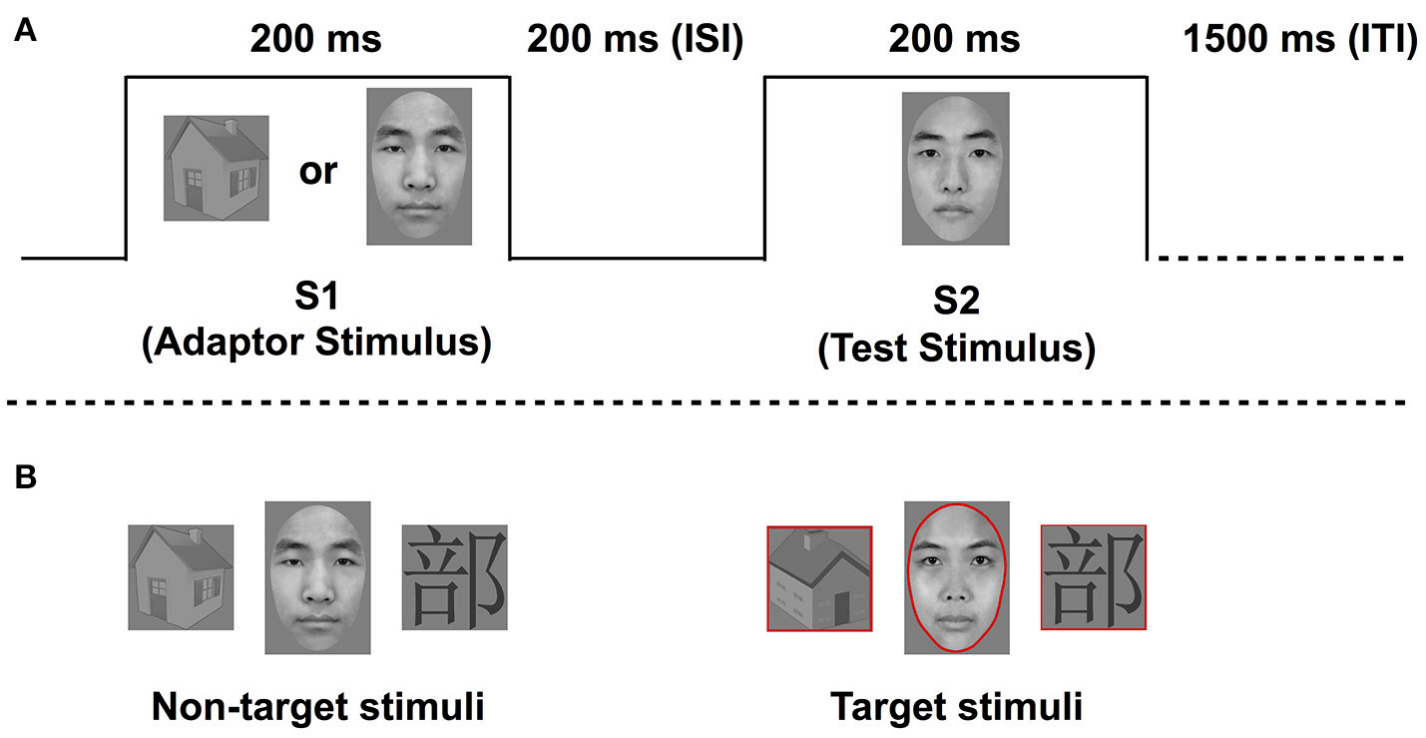
The example of the trial sequence of face adaptation **(A)** and the example of target and non-target stimuli for face/word processing **(B)**.

In each trial, an adaptor and test stimulus were presented sequentially for 200 ms each, with an ISI of 200 ms, followed by a 1,500 ms intertrial interval (see Figure 1), similarly to Eimer et al. (2010). The two possible types of test stimuli were faces (F) and Chinese characters (C). Each test stimulus was preceded by 1 of 2 possible adaptor stimuli, either from the same category or from a non-expert category (houses; H) as the control condition. Specifically, a face was preceded by a face or a house, and a Chinese character was preceded by a Chinese character or a house. Therefore, there were 4 conditions, including FF, HF, CC, and HC. The conditions were presented with equal frequency, and in a random order within each block. There were 72 trials in each block, 64 of which were non-target trials. No response was required during the non-target trials. The remaining 8 trials per block were target trials, where a red outline shape aligned with the outer contours of the stimulus shape. The aim of the target trials was for participants to focus their attention on the task. The target stimulus, namely the red outline shape, was presented with equal probability as the study or test stimulus in the target trials. Target trials were randomly intermixed with non-target trials. There were four blocks. Participants were instructed to press a response button when they detected a target stimulus, following the second picture presentation.

### Electroencephalographic recordings and data analyses

Electroencephalography (EEG) was performed using a 128-channel HydroCel Geodesic Sensor Net (Electrical Geodesics, Inc., Eugene, OR, USA), with an electrode placed on the Vertex (Cz) serving as the reference for the online recording. Electrode impedances were maintained below 50 kΩ. Signals were digitized at a 500 Hz sampling rate, and amplified with a 0.1–200 Hz elliptical band pass filter. EEG data were digitally filtered offline using a 0.1–30 Hz band pass filter and epoched from 200 ms before to 800 ms after stimuli onset, with baseline at 100 ms before stimulus onset. Trials with artifacts exceeding ±100 μV were rejected. Accepted trials were categorized by condition and participant (mean, 58.9; range, 50–64). The remaining EEG data were re-referenced to the average of the channels.

EEG data were analyzed for non-target trials only. A group of channels over the left occipitotemporal regions (O1, 65, T5; channel 65, between O1 and T5) and right occipitotemporal regions (O2, 90, T6; channel 90, between O2 and T6) were analyzed when the N170 components were maximal (Cao et al., 2014a). To increase reliability, these peak amplitudes were then averaged across the 3 channels for each hemisphere. EEG waveforms were averaged separately for each presentation condition. Visual inspection of the individual data and the results of previous studies (Eimer et al., 2010; Nemrodov and Itier, 2011; Cao et al., 2015a) were used to define the N170 time-window as 130–210 ms for adaptor stimuli and 140–220 ms for test stimuli.

Previous studies have reported that the P100 amplitude elicited by faces is different from that elicited by words (Mercure et al., 2011; Fu et al., 2012; Cao et al., 2015b). In order to ensure that N170 amplitude differences could not be attributed to P100 amplitude differences between faces and Chinese characters, statistical analyses of the N170 amplitude focused on peak-to-peak measurements, for which baseline-to-peak P100 amplitudes were subtracted from baseline-to-peak N170 amplitudes (Itier and Taylor, 2002; Goffaux et al., 2003; Rossion et al., 2003; Bentin et al., 2007; Tian et al., 2015). Statistical analyses of the baseline-to-peak N170 amplitude are reported in the Appendix.

N170 responses evoked by the adaptor stimuli were analyzed using repeated-measures analysis of variance (ANOVA), with factors including stimulus category (F, C, and H), hemisphere (left, right), and sex (women, men). The N170 responses elicited by the test stimuli (F and C) were also analyzed using ANOVA for the test category (F and C), paired condition (within category, FF/CC; control category, HF/HC), hemisphere (left or right), and sex (women, men). In order to directly understand adaptation differences evoked by the test stimuli (e.g., F or C) between women and men, the analysis was also performed on the adaptation index (AI). The face AI was defined as F_index = (HF − FF)/(HF + FF) and the Chinese character AI was defined as C_index = (HC − CC)/(HC + CC). The AI was also analyzed using ANOVA for test category (F, C), hemisphere (left, right), and sex (women, men). All *post-hoc* paired comparisons were performed using the Bonferroni adjustment for multiple comparisons, and an alpha level of 0.05. We also examined the correlation between the N170 AI for facial processing and for Chinese characters, in both women and men.

## Results

### Behavioral results

The mean accuracy of detecting the target stimuli was 0.97 (standard deviation, SD = 0.023). The mean response time was 533 ms (SD = 17.15). There were no main effects or interactions.

### ERP results

#### Adaptor stimuli N170 amplitudes

**Figure 2:**
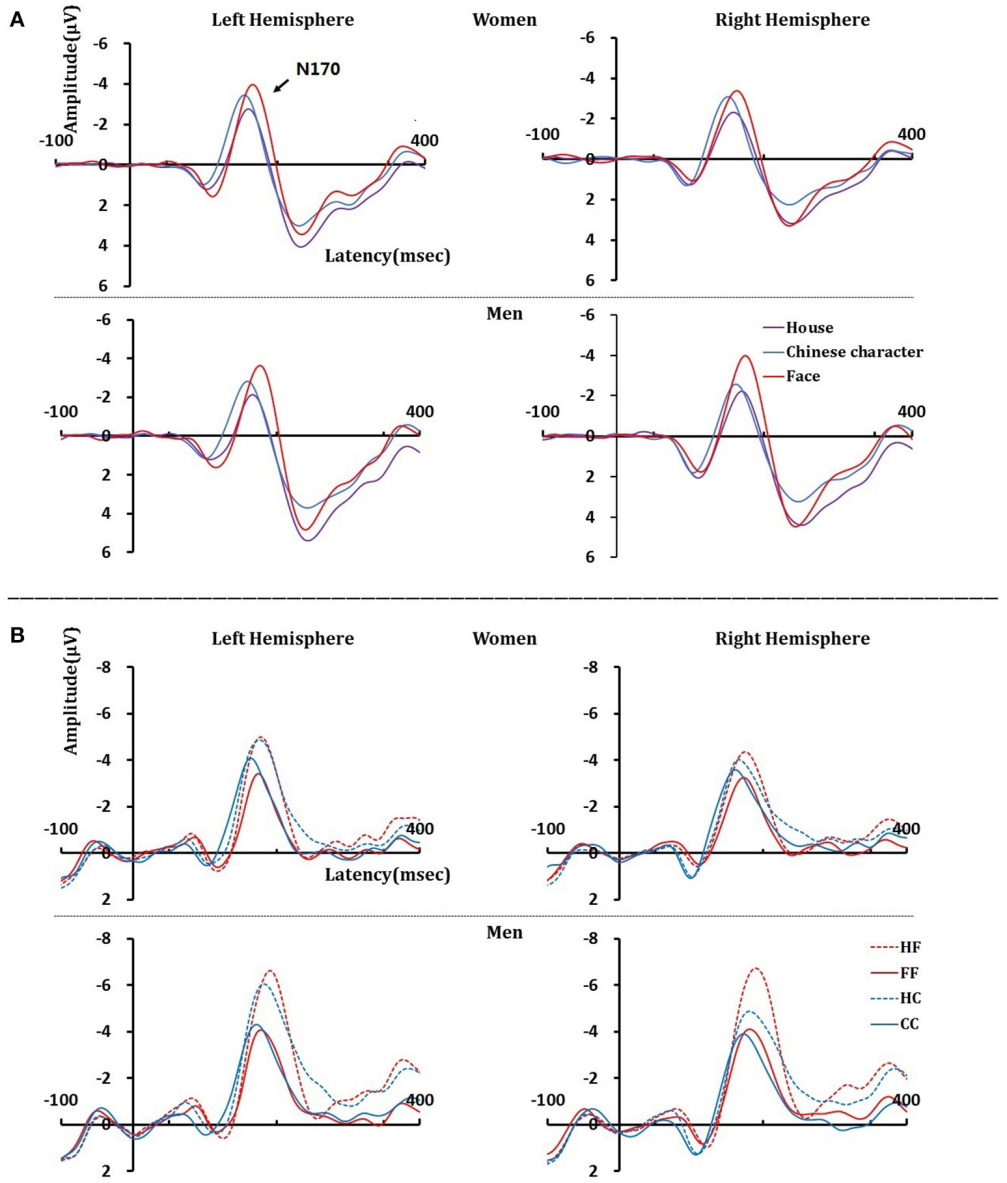
N170 component at the left and right hemispheres elicited by study **(A)** and test **(B)** stimuli in women and men.

The results are shown in Figure 2 and Table 1. The analysis of the peak-to-peak N170 amplitudes yielded a significant main effect for the stimulus category, *F*_(2, 136)_ = 84.588, *p* < 0.001, η^2^_*p*_ = 0.554, with larger N170 amplitudes elicited by faces than by Chinese characters, *t*_(69)_ = 6.806, *p* < 0.001, or houses, *t*_(69)_ = 13.724, *p* < 0.001. Furthermore, N170 amplitudes for Chinese characters were significantly larger than those for houses, *t*_(69)_ = 5.307, *p* < 0.001. There was also a trend toward a stimulus category × hemisphere × sex interaction, *F*_(2, 136)_ = 3.047, *p* = 0.063, η^2^_*p*_ = 0.043. Further analyses revealed that when faces were used as the adaptor stimuli, there was a significant main effect for sex *F*_(1, 68)_ = 5.841, *p* = 0.018, η^2^_*p*_ = 0.079, and a trend toward an interaction between hemisphere and sex, *F*_(1, 68)_ = 3.821, *p* = 0.055, η^2^_*p*_ = 0.053. *Post-hoc t*-tests revealed that the N170 amplitude elicited by faces was right-lateralized in men, *t*_(35)_ = 2.230, *p* = 0.032, and bilateral in women, *t*_(33)_ = 0.737, *p* = 0.466. Moreover, when the adaptor stimuli were Chinese characters or houses, no main effects or interactions were observed.

**Table 1:** The peak-to-peak N170 amplitude(μV, M ± SD) elicited by the adaptor stimuli.

	**Female**		**Male**	
	**Left hemisphere**	**Right hemisphere**	**Left hemisphere**	**Right hemisphere**
Face	−6.44 ± 3.55	−6.07 ± 3.42	−7.73 ± 3.07	−8.59 ± 4.07
Chinese character	−5.62 ± 3.03	−5.23 ± 2.99	−6.42 ± 3.07	−6.08 ± 3.7
House	−4.57 ± 2.76	−4.28 ± 2.67	−4.95 ± 3.1	−5.46 ± 3.4

#### Test stimuli N170 amplitudes

The results are shown in Figure 2 and Table 2. Repeated measures ANOVA of N170 amplitudes, including test category, paired condition, hemisphere, and sex revealed significant main effects for test category, *F*_(1, 68)_ = 6.944, *p* = 0.010, η^2^_*p*_ = 0.093, paired condition, *F*_(1, 68)_ = 248.738, *p* < 0.001, η^2^_*p*_ = 0.785, and sex, *F*_(1, 68)_ = 9.061, *p* = 0.004, η^2^_*p*_ = 0.118. Furthermore, there was a trend toward an interaction for test category × paired condition × hemisphere × sex, *F*_(1, 68)_ = 3.546, *p* = 0.064, η^2^_*p*_ = 0.050. The following significant interactions also occurred: test category × paired condition × hemisphere, *F*_(1, 68)_ = 10.296, *p* = 0.002, η^2^_*p*_ = 0.132; test category × hemisphere × sex, *F*_(1, 68)_ = 4.392, *p* = 0.040, η^2^_*p*_ = 0.061; paired condition × sex, *F*_(1, 68)_ = 12.814, *p* = 0.001, η^2^_*p*_ = 0.159; test category × paired condition, *F*_(1, 68)_ = 64.352, *p* < 0.001, η^2^_*p*_ = 0.486; and test category × hemisphere, *F*_(1, 68)_ = 9.397, *p* = 0.003, η^2^_*p*_ = 0.121.

**Table 2:** The peak-to-peak N170 amplitude(μV, M ± SD) elicited by the test stimuli.

	**Female**		**Male**	
	**Left hemisphere**	**Right hemisphere**	**Left hemisphere**	**Right hemisphere**
FF	−4.42 ± 2.58	−4.34 ± 2.72	−5.81 ± 2.82	−6.68 ± 4.15
HF	−7.13 ± 3.85	−6.99 ± 4.16	−9.48 ± 3.08	−10.59 ± 4.56
CC	−5.1 ± 2.8	−4.91 ± 2.78	−6.25 ± 2.76	−6.38 ± 3.26
HC	−6.4 ± 3.73	−5.92 ± 3.57	−8.89 ± 3.15	−8.34 ± 3.92

Three-way ANOVAs for paired condition, hemisphere, and sex were conducted separately for face and Chinese character stimuli. For the face test stimuli, a significant paired condition × sex interaction, *F*_(1, 68)_ = 7.918, *p* = 0.006, η^2^_*p*_ = 0.104, occurred; *post-hoc t*-tests revealed that the N170 amplitude was smaller when preceded by within-category stimuli than by when preceded by control category stimuli in both women, *t*_(33)_ = 9.919, *p* < 0.001, and men, *t*_(35)_ = 13.272, *p* < 0.001. There was also a trend toward an interaction between hemisphere and sex, *F*_(1, 68)_ = 3.195, *p* = 0.078, η^2^_*p*_ = 0.045; *post-hoc t*-tests revealed that the N170 amplitude was right-lateralized in men*, t*_(68)_ = 2.580, *p* = 0.014), and bilateral in women.

For Chinese characters, a significant main effect for paired condition was observed, *F*_(1, 68)_ = 105.272, *p* < 0.001, η^2^_*p*_ = 0.608, and there was also a significant paired condition × hemisphere interaction, *F*_(1, 68)_ = 9.570, *p* = 0.003, η^2^_*p*_ = 0.123. *Post-hoc t*-tests revealed that, in both hemispheres, the N170 amplitude was larger when preceded by house stimuli than when preceded by Chinese characters [left: *t*_(69)_ = 9.713, *p* < 0.001; right: *t*_(69)_ = 7.859, *p* < 0.001]. There was also a significant paired condition × sex interaction, *F*_(1, 68)_ = 11.537, *p* = 0.001, η^2^_*p*_ = 0.145; *post-hoc t*-tests revealed that N170 amplitudes were larger in men than in women in both paired conditions [CC, *t*_(68)_ = 2.066, *p* = 0.043; HC, *t*_(68)_ = 3.129, *p* = 0.003].

#### Test stimuli N170 amplitude AI

The analysis of N170 amplitude AIs revealed a significant main effect of sex, *F*_(1, 68)_ = 4.711, *p* = 0.033, η^2^_*p*_ = 0.065, with lower AIs for women (0.17) compared to men (0.21). A significant main effect of test category, *F*_(1, 68)_ = 58.389, *p* < 0.001, η^2^_*p*_ = 0.462, occurred, with greater AIs for faces (0.25) than for Chinese characters (0.13). There was also a significant test category × hemisphere interaction, *F*_(1, 68)_ = 4.882, *p* = 0.031, η^2^_*p*_ = 0.067; *post-hoc t-*tests revealed that the AI in the left hemisphere was greater than that in the right hemisphere for Chinese characters, *t*_(69)_ = 2.685, *p* = 0.009. Furthermore, the AI for faces was greater than that for Chinese characters in both hemispheres [left: *t*_(69)_ = 4.788, *p* < 0.001; right: *t*_(69)_ = 7.231, *p* < 0.001).

#### Correlation analyses

**Figure 3:**
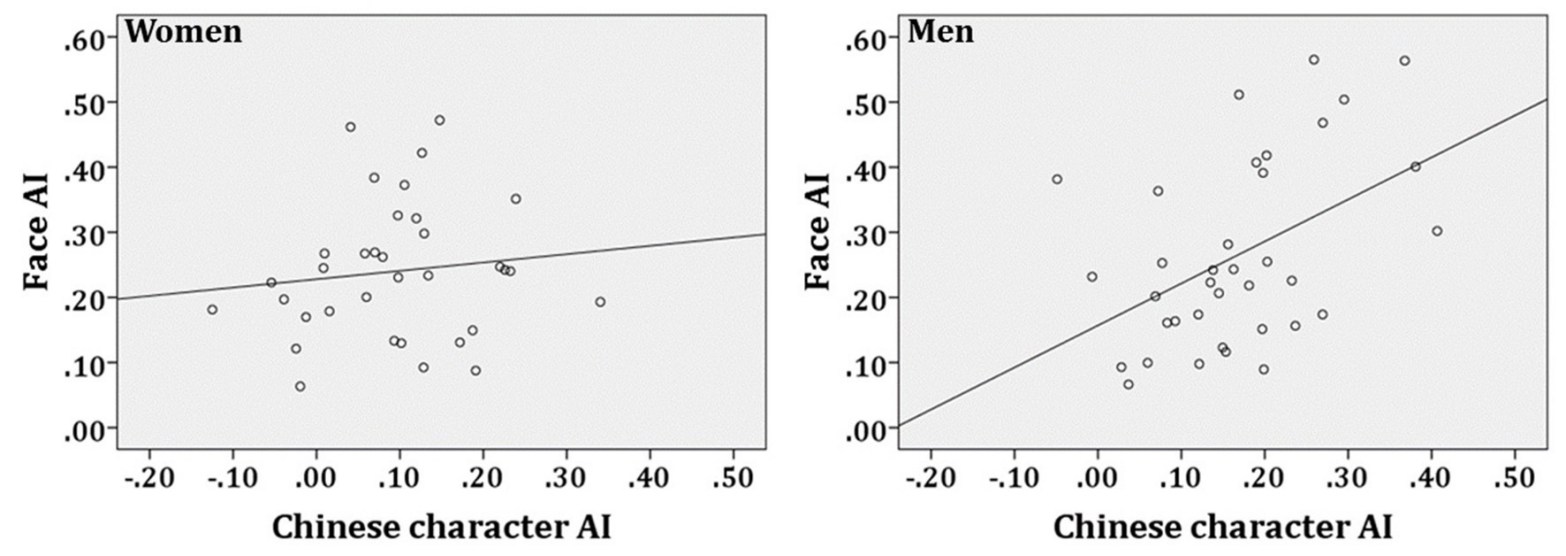
Correlations of the N170 adaptation level between faces and Chinese characters in women and men. AI refers to Adaptation Index.

The face N170 AI was defined as the F_index, and the Chinese character N170 AI was defined as the C_index. Correlational analyses of AI between faces and Chinese characters revealed a significant correlation in men, *r* = 0.467, *p* = 0.004, although this correlation was not observed in women, *r* = 0.121, *p* = 0.495 (see Figure 3).

## Discussion

The present study was conducted to investigate sex differences in N170 categorical adaptation during early perceptual processing of faces and Chinese characters. Recent studies have confirmed that the N170 amplitude is strongly suppressed when a target stimulus is preceded by another within-category stimulus, compared to when it is preceded by an image from a different object category (Kovács et al., 2005; Cao et al., 2014a, 2015a,b). This repetition effect is referred to as categorical adaptation, and has been consistently demonstrated across studies (Eimer et al., 2010, 2011; Kloth et al., 2010; Walther et al., 2013). In the present study, we used an N170 adaptation paradigm to assess whether categorical adaptation differs between women and men during face and Chinese character processing. Moreover, the present study produced 2 improvements to the accuracy of categorical adaptation measurements. First, the N170 amplitude value in the present study is the peak-to-peak measurement, for which the baseline-to-peak P100 amplitude was subtracted from the baseline-to-peak N170 amplitude. In the present study, the P100 amplitude elicited by faces differed from that elicited by Chinese characters (see Appendix), which is consistent with many previous studies (Maurer et al., 2008; Fu et al., 2012; Cao et al., 2015b). Thus, peak-to-peak N170 amplitude measurements may reduce the influence of low-level stimulus properties on N170 effects related to faces and Chinese characters. Second, the N170 categorical AI in the present study was a pure numerical index; different categories of objects could be directly compared.

In the present study, we assessed sex differences in categorical adaptation during face and Chinese character processing; we discussed the neural response to adaptor stimuli between women and men, and discussed the detailed neural response to test stimuli between women and men. For the adaptor stimuli, the N170 amplitude elicited by expert stimuli was larger than that elicited by houses in both women and men. Furthermore, the N170 amplitude elicited by faces as adaptor stimuli was larger in the right hemisphere than in the left hemisphere in men, although it was similar in both hemispheres in women; these results are also consistent with those of previous studies (Proverbio et al., 2010; Ji et al., 2016). Together, our results and previous studies support that the N170 response during face processing is right hemisphere-dominant in men and bilateral in women; furthermore, this effect is stable across different tasks (for example, the one-back task and adaptation task). In contrast, the N170 amplitude elicited by Chinese characters as adaptor stimuli is bilateral in women and men. Previous studies have found that the N170 amplitude elicited by Chinese characters was bilateral in men, whereas it was left lateralized in the one-back task in women (Ji et al., 2016). Therefore, women may have more flexible processing styles than men during Chinese character recognition tasks.

### Sex differences in N170 categorical adaptation

The N170 categorical adaptation analysis indicated 3 significant differences between women and men. First, the present results are the first demonstration that the N170 categorical AI is greater in men than in women, for both face and Chinese character processing. The results support sex differences in early perceptual processing for objects of expertise. Many studies have demonstrated that N170 responses during face and word processing differ between women and men (Proverbio et al., 2010; Ji et al., 2016). Our findings support sex differences in categorical adaptation, which is a reduction in neural activity in stimulus-sensitive neuron populations following stimulus repetition, for expert object processing. Moreover, although the present study does not resolve the debate on the nature of categorical adaptation, it does contribute to knowledge regarding the adaptation mechanisms. Although several studies have investigated the neural mechanisms of categorical adaptation, the nature of this phenomenon remains unclear. Many studies have connected categorical adaptation to local or entirely bottom-up mechanisms (e.g., fatigue, sharpening, or response facilitation; for a review see Grill-Spector et al., 2006). Previous evidence suggests that women perform better than men on both face and word recognition tasks (Messé et al., 1968; May and Hutt, 1974; Godard and Fiori, 2010; Herlitz and Lovén, 2013); the present study found that men demonstrated improved adaptation compared to women. Although a correlation between processing performance and the degree of adaptation for expert stimuli processing was not directly assessed, the findings described above imply a negative correlation. Further explorations of this possibility may support fatigue mechanisms of categorical adaptation. Although single-cell recording studies suggest that a single fatigue-related adaptation cannot accurately describe adaptation-related phenomena (Vogels, 2016), future studies should directly investigate the relationship between behavioral performance for face/word processing and the degree of categorical adaptation in both women and men.

The second difference between women and men revealed by our study was the significant correlation for the N170 categorical AI between face and Chinese character processing in men, which was not observed in women. The results indicate that men may have similar N170 categorical adaptation traits for faces and Chinese characters, whereas women may process different categories of objects of expertise differently. Many previous studies support interactions between face and word processing (Nestor et al., 2013; Dehaene et al., 2015), particularly lesion case studies (e.g., prosopagnosia/pure alexia). For example, Behrmann and colleagues found that patients with prosopagnosia had impairments reading words (Behrmann and Kimchi, 2003; Behrmann and Plaut, 2014) and pure alexia with face impairments (Behrmann and Plaut, 2014). Furthermore, both patients in Behrmann and Kimchi (2003), and 6 of the 7 patients in Behrmann and Plaut (2014) were men. Together, the results of the present and previous studies suggest that interactions between face and word processing may occur only in men. Future research should examine this possibility.

The third N170 categorical adaptation difference between women and men is lateralization of the N170 amplitude evoked by test faces. In the present study, the N170 amplitude elicited by test faces was right lateralized in men, and was bilateral in women. These lateralization differences for face processing between women and men were also found in previous studies. For example, Proverbio et al. (2010) found bilateral N170 responses in women, and right lateralized responses in men, when processing faces representing different ages and genders. Our results are consistent with previous findings, and also support that the phenomenon is consistent for processing repeated face stimuli.

### Similarities between men and women in N170 categorical adaptation

Previous studies have supported stable N170 categorical adaptation for both face and word recognition (Cao et al., 2014b, 2015b). The present study demonstrated that the N170 responses to faces/Chinese characters are significantly greater in control trials compared to within-category trials, in both women and men. Our study extends the current knowledge by demonstrating that N170 categorical adaptation for faces and Chinese characters is consistent in both sexes.

We found several similarities in N170 categorical adaptation between women and men, for both face and word processing. First, the N170 categorical AI is greater for face processing than for Chinese characters in both sexes. Previous studies have reported a difference in N170 categorical adaptation between faces and words (Mercure et al., 2011). Our results, which present the N170 categorical AI as a pure numerical index for the first time, directly demonstrate that N170 categorical adaptation is greater for faces than for Chinese characters. Therefore, N170 categorical adaptation may be associated with common configuration within a category (Mercure et al., 2011). Face-related N170 is thought to reflect structural encoding of faces (Bentin and Deouell, 2000; Eimer, 2000b). Since all faces have the same first-order configuration of 2 eyes above a nose, which is above a mouth (Maurer et al., 2002), structural encoding processing may affect the configuration of subsequent faces, resulting in reduced N170 responses. In contrast, previous studies have demonstrated that word-related N170 effects may reflect visual word form recognition (Maurer et al., 2005a). Different words share fewer common configurations than faces (Mercure et al., 2011); therefore, N170 categorical adaptation may be lower for words than that for faces.

The present results indicated a second similarity between women and men; the N170 categorical AI for face processing was similar between hemispheres, whereas it was left lateralized for Chinese character processing. These findings suggest that the right hemisphere has similar characteristics to the left hemisphere for perceptual processing of repeated faces, whereas the hemispheres have different characteristics for Chinese character processing. Although there were some differences in the experimental paradigm, previous studies have demonstrated that there are different N170 habituation properties between face and word processing. For example, Fu and colleagues found that a left-lateralized N170 effect occurred for Chinese character processing, and did not occur for face processing (Fu et al., 2012; Feng et al., 2013). Our results support consistency in bilateral N170 categorical adaptation for faces and left-lateralized effects for Chinese characters, in both sexes.

Together, our results reveal that men have greater N170 categorical adaptation for face and word processing than women have. Furthermore, there is a significant correlation between the N170 categorical AIs for face and Chinese character processing in men, which is not observed in women. These findings suggest that the categorical adaptation characteristics of men differ from those of women, for processing repeated faces or words. However, only 2 categories of objects of expertise were used in the present study, and it is therefore not clear whether sex differences in N170 categorical adaptation also occur for other objects of expertise (e.g., cars). Moreover, the Chinese characters were familiar to participants, whereas the face images were unfamiliar; we did not use different levels of face and Chinese character familiarity to directly explore the influence of familiarity on sex differences. Future studies should examine whether familiarity with expert stimuli influences sex differences in N170 categorical adaptation for faces and Chinese characters.

## Author contributions

CZ and XC: designed the experiments; LJ, SC, and XC: wrote the manuscript; CZ and XM: executed the project; CZ, XM, and XC: performed the data analysis; All authors reviewed the manuscript.

### Conflict of interest statement

The authors declare that the research was conducted in the absence of any commercial or financial relationships that could be construed as a potential conflict of interest.

## References

[B1] Behrmann M. Kimchi R. (2003). What does visual agnosia tell us about perceptual organization and its relationship to object perception. J. Exp. Psychol. 29, 19–42. 10.1037/0096-1523.29.1.19 12669745

[B2] Behrmann M. Plaut D. C. (2014). Bilateral hemispheric processing of words and faces: evidence from word impairments in prosopagnosia and face impairments in pure alexia. Cereb. Cortex 24, 1102–1118. 10.1093/cercor/bhs390 23250954

[B3] Bentin S. Allison T. Puce A. Perez E. McCarthy G. (1996). Electrophysiological studies of face perception in humans. J. Cogn. Neurosci. 8, 551–565. 10.1162/jocn.1996.8.6.551 20740065 PMC2927138

[B4] Bentin S. Deouell L. Y. (2000). Structural encoding and identification in face processing: ERP evidence for separate mechanisms. Cogn. Neuropsychol. 17, 35–55. 10.1080/026432900380472 20945170

[B5] Bentin S. Mouchetant-Rostaing Y. Giard M. H. Echallier J. F. Pernier J. (1999). ERP manifestations of processing printed words at different psycholinguistic levels: time course and scalp distribution. J. Cogn. Neurosci. 11, 235–260. 10.1162/089892999563373 10402254

[B6] Bentin S. Taylor M. J. Rousselet G. A. Itier R. J. Caldara R. Schyns P. G.. (2007). Controlling interstimulus perceptual variance does not abolish N170 face sensitivity. Nat. Neurosci. 10, 801–802. 10.1038/nn0707-801 17593935

[B7] Cao X. Jiang B. Li C. He Z. (2014a). Rapid adaptation effect of N170 for printed words. Percept. Mot. Skills 119, 191–202. 10.2466/24.22.PMS.119c15z6 25153749

[B8] Cao X. Jiang B. Li C. Xia N. Floyd R. J. (2015a). The commonality between the perceptual adaptation mechanisms involved in processing faces and nonface objects of expertise. Neuropsychology 29, 715–725. 10.1037/neu0000170 25643216

[B9] Cao X. Li S. Zhao J. Lin S. Weng X. (2011). Left-lateralized early neurophysiological response for Chinese characters in young primary school children. Neurosci. Lett. 492, 165–169. 10.1016/j.neulet.2011.02.002 21310213

[B10] Cao X. Zhang H. (2011). Change in subtle N170 specialization in response to Chinese characters and pseudocharacters. Percept. Mot. Skills 113, 365–376. 10.2466/04.22.24.28.PMS.113.5.365-376 22185051

[B11] Cao X. Jiang B. Gaspar C. Li C. (2014b). The overlap of neural selectivity between faces and words: evidences from the N170 adaptation effect. Exp. Brain Res. 232, 3015–3021. 10.1007/s00221-014-3986-x 24854017

[B12] Cao X. Ma X. Qi C. (2015b). N170 adaptation effect for repeated faces and words. Neuroscience 294, 21–28. 10.1016/j.neuroscience.2015.03.009 25772788

[B13] Cohen L. Dehaene S. (2004). Specialization within the ventral stream: the case for the visual word form area. Neuroimage 22, 466–476. 10.1016/j.neuroimage.2003.12.049 15110040

[B14] Dehaene S. Cohen L. Morais J. Kolinsky R. (2015). Illiterate to literate: behavioural and cerebral changes induced by reading acquisition. Nat. Rev. Neurosci. 16, 234–244. 10.1038/nrn3924 25783611

[B15] Eimer M. (2000a). Effects of face inversion on the structural encoding and recognition of faces: evidence from event-related brain potentials. Cogn. Brain Res. 10, 145–158. 10.1016/S0926-6410(00)00038-0 10978702

[B16] Eimer M. (2000b). Event-related brain potentials distinguish processing stages involved in face perception and recognition. Clin. Neurophysiol. 111, 694–705. 10.1016/S1388-2457(99)00285-0 10727921

[B17] Eimer M. Gosling A. Nicholas S. Kiss M. (2011). The N170 component and its links to configural face processing: a rapid neural adaptation study. Brain Res. 1376, 76–87. 10.1016/j.brainres.2010.12.046 21172312

[B18] Eimer M. Kiss M. Nicholas S. (2010). Response profile of the face-sensitive N170 component: a rapid adaptation study. Cereb. Cortex 20, 2442–2452. 10.1093/cercor/bhp312 20080930

[B19] Feng C. Luo Y. Fu S. (2013). The category-sensitive and orientation-sensitive N170 adaptation in faces revealed by comparison with Chinese characters. Psychophysiology 50, 885–899. 10.1111/psyp.12067 23802879

[B20] Fu S. Feng C. Guo S. Luo Y. Parasuraman R. (2012). Neural adaptation provides evidence for categorical differences in processing of faces and Chinese characters: an ERP study of the N170. PLoS ONE 7:e41103. 10.1371/journal.pone.0041103 22911750 PMC3404057

[B21] Godard O. Fiori N. (2010). Sex differences in face processing: are women less lateralized and faster than men? Brain Cogn. 73, 167–175. 10.1016/j.bandc.2010.04.008 20621740

[B22] Godard O. Leleu A. Rebaï M. Fiori N. (2013). Sex differences in interhemispheric communication during face identity encoding: evidence from ERPs. Neurosci. Res. 76, 58–66. 10.1016/j.neures.2013.03.005 23524245

[B23] Goffaux V. Gauthier I. Rossion B. (2003). Spatial scale contribution to early visual differences between face and object processing. Cogn. Brain Res. 16, 416–424. 10.1016/S0926-6410(03)00056-9 12706221

[B24] Grill-Spector K. Henson R. Martin A. (2006). Repetition and the brain: neural models of stimulus-specific effects. Trends Cogn. Sci. 10, 14–23. 10.1016/j.tics.2005.11.006 16321563

[B25] Herlitz A. Lovén J. (2013). Sex differences and the own-gender bias in face recognition: a meta-analytic review. Vis. Cogn. 21, 1306–1336. 10.1080/13506285.2013.823140

[B26] Hill H. Ott F. Herbert C. Weisbrod M. (2006). Response execution in lexical decision tasks obscures sex-specific lateralization effects in language processing: evidence from event-related potential measures during word reading. Cereb. Cortex 16, 978–989. 10.1093/cercor/bhj040 16177269

[B27] Itier R. J. Taylor M. J. (2002). Inversion and contrast polarity reversal affect both encoding and recognition processes of unfamiliar faces: a repetition study using ERPs. Neuroimage 15, 353–372. 10.1006/nimg.2001.0982 11798271

[B28] Itier R. J. Taylor M. J. (2004). N170 or N1? Spatiotemporal differences between object and face processing using ERPs. Cereb. Cortex 14, 132–142. 10.1093/cercor/bhg111 14704210

[B29] Ji L. Cao X. Xu B. (2016). Sex differences of hemispheric lateralization for faces and Chinese characters in early perceptual processing. Neurosci. Lett. 635, 77–82. 10.1016/j.neulet.2016.10.037 27777136

[B30] Kanwisher N. McDermott J. Chun M. M. (1997). The fusiform face area: a module in human extrastriate cortex specialized for face perception. J. Neurosci. 17, 4302–4311. 9151747 10.1523/JNEUROSCI.17-11-04302.1997 PMC6573547

[B31] Kloth N. Schweinberger S. R. Kovács G. (2010). Neural correlates of generic versus gender-specific face adaptation. J. Cogn. Neurosci. 22, 2345–2356. 10.1162/jocn.2009.21329 19702459

[B32] Kovács G. Zimmer M. Bankó É. Harza I. Antal A. Vidnyánszky Z. (2005). Electrophysiological correlates of visual adaptation to faces and body parts in humans. Cereb. Cortex 16, 742–753. 10.1093/cercor/bhj020 16120795

[B33] Lin S.-E. Chen H.-C. Zhao J. Li S. He S. Weng X.-C. (2011). Left-lateralized N170 response to unpronounceable pseudo but not false Chinese characters—the key role of orthography. Neuroscience 190, 200–206. 10.1016/j.neuroscience.2011.05.071 21704128

[B34] Maurer D. Grand R. L. Mondloch C. J. (2002). The many faces of configural processing. Trends Cogn. Sci. 6, 255–260. 10.1016/S1364-6613(02)01903-4 12039607

[B35] Maurer U. Brandeis D. McCandliss B. D. (2005a). Fast, visual specialization for reading in English revealed by the topography of the N170 ERP response. Behav. Brain Funct. 1:13. 10.1186/1744-9081-1-13 16091138 PMC1208852

[B36] Maurer U. Brem S. Bucher K. Brandeis D. (2005b). Emerging neurophysiological specialization for letter strings. J. Cogn. Neurosci. 17, 1532–1552. 10.1162/089892905774597218 16269095

[B37] Maurer U. Rossion B. McCandliss B. D. (2008). Category specificity in early perception: face and word n170 responses differ in both lateralization and habituation properties. Front. Hum. Neurosci. 2:18. 10.3389/neuro.09.018.2008 19129939 PMC2614860

[B38] May R. B. Hutt C. (1974). Modality and sex differences in recall and recognition memory. Child Dev. 228–231. 10.2307/1127777 4820280

[B39] Mercure E. Cohen Kadosh K. Johnson M. (2011). The N170 shows differential repetition effects for faces, objects, and orthographic stimuli. Front. Hum. Neurosci. 5:6. 10.3389/fnhum.2011.00006 21283529 PMC3031024

[B40] Messé L. A. Chisena P. R. Shipley R. H. (1968). A sex difference in the recognition level of words. Psychon. Sci. 11, 131–132. 10.3758/BF03331008

[B41] Nemrodov D. Itier R. J. (2011). The role of eyes in early face processing: a rapid adaptation study of the inversion effect. Br. J. Psychol. 102, 783–798. 10.1111/j.2044-8295.2011.02033.x 21988384 PMC3933317

[B42] Nestor A. Behrmann M. Plaut D. C. (2013). The neural basis of visual word form processing: a multivariate investigation. Cereb. Cortex 23, 1673–1684. 10.1093/cercor/bhs158 22693338 PMC13377679

[B43] Proverbio A. M. Brignone V. Matarazzo S. Del Zotto M. Zani A. (2006). Gender differences in hemispheric asymmetry for face processing. BMC Neurosci. 7:44. 10.1186/1471-2202-7-44 16762056 PMC1523199

[B44] Proverbio A. M. Mazzara R. Riva F. Manfredi M. (2012). Sex differences in callosal transfer and hemispheric specialization for face coding. Neuropsychologia 50, 2325–2332. 10.1016/j.neuropsychologia.2012.05.036 22727879

[B45] Proverbio A. M. Riva F. Martin E. Zani A. (2010). Face coding is bilateral in the female brain. PLoS ONE 5:e11242. 10.1371/journal.pone.0011242 20574528 PMC2888585

[B46] Proverbio A. M. Riva F. Zani A. Martin E. (2011). Is it a baby? Perceived age affects brain processing of faces differently in women and men. J. Cogn. Neurosci. 23, 3197–3208. 10.1162/jocn_a_00041 21557651

[B47] Richler J. J. Gauthier I. (2014). A meta-analysis and review of holistic face processing. Psychol. Bull. 140, 1281–1302. 10.1037/a0037004 24956123 PMC4152424

[B48] Rossion B. Jacques C. (2008). Does physical interstimulus variance account for early electrophysiological face sensitive responses in the human brain? Ten lessons on the N170. Neuroimage 39, 1959–1979. 10.1016/j.neuroimage.2007.10.011 18055223

[B49] Rossion B. Joyce C. A. Cottrell G. W. Tarr M. J. (2003). Early lateralization and orientation tuning for face, word, and object processing in the visual cortex. Neuroimage 20, 1609–1624. 10.1016/j.neuroimage.2003.07.010 14642472

[B50] Skrandies W. Reik P. Kunze C. (1999). Topography of evoked brain activity during mental arithmetic and language tasks: sex differences. Neuropsychologia 37, 421–430. 10.1016/S0028-3932(98)00103-1 10215089

[B51] Sun Y. Gao X. Han S. (2010). Sex differences in face gender recognition: an event-related potential study. Brain Res. 1327, 69–76. 10.1016/j.brainres.2010.02.013 20153301

[B52] Tian T. Feng X. Feng C. Gu R. Luo Y.-J. (2015). When rapid adaptation paradigm is not too rapid: evidence of face-sensitive N170 adaptation effects. Biol. Psychol. 109, 53–60. 10.1016/j.biopsycho.2015.03.011 25888326

[B53] Vogels R. (2016). Sources of adaptation of inferior temporal cortical responses. Cortex 80, 185–195. 10.1016/j.cortex.2015.08.024 26518166

[B54] Walther C. Schweinberger S. R. Kaiser D. Kovács G. (2013). Neural correlates of priming and adaptation in familiar face perception. Cortex 49, 1963–1977. 10.1016/j.cortex.2012.08.012 23021070

[B55] Wirth M. Horn H. Konig T. Stein M. Federspiel A. Meier B.. (2007). Sex differences in semantic processing: event-related brain potentials distinguish between lower and higher order semantic analysis during word reading. Cereb. Cortex 17, 1987–1997. 10.1093/cercor/bhl121 17116651

[B56] Yum Y. N. Holcomb P. J. Grainger J. (2011). Words and pictures: an electrophysiological investigation of domain specific processing in native Chinese and English speakers. Neuropsychologia 49, 1910–1922. 10.1016/j.neuropsychologia.2011.03.018 21439991 PMC3100363

[B57] Zhao J. Li S. Lin S. E. Cao X. H. He S. Weng X. C. (2012). Selectivity of N170 in the left hemisphere as an electrophysiological marker for expertise in reading Chinese. Neurosci. Bull. 28, 577–584. 10.1007/s12264-012-1274-y 23054635 PMC5561926

