# Supplementary material for: Sex Differences in Categorical Adaptation for Faces and Chinese Characters during Early Perceptual Processing
Source: Front Hum Neurosci. 2018 Jan 12;11:656. doi: 10.3389/fnhum.2017.00656 (PMC5770371; doi:10.3389/fnhum.2017.00656)
Supplement: Supplementary file 1 [file DataSheet1.pdf]

Appendix : Supplementary data

The results of the peak amplitude for P100 and N170 components were as followed:

1 Adaptor stimuli results

The ERP responses elicited by study stimuli were analyzed in a repeated-measures analysis of variance (ANOVA) for the factors Stimulus Category [Face, Chinese character and House], Hemisphere (Left, Right) and Sex (Women, Men). The results are shown in Table 3.

Table 3. The p100 and baseline-to-peak N170 amplitude (μV, M（SD）) elicited by the adaptor stimuli

		Face		Chinese character		House	
		LH	RH	LH	RH	LH	RH
Female	P100	1.8 (1.12)	1.72 (1.19)	1.53 (1.24)	1.68 (1.38)	1.72 (1.2)	1.69 (1.31)
	N170	-4.64 (2.99)	-4.35 (2.65)	-4.1 (2.48)	-3.56 (2.34)	-2.84 (2.24)	-2.59 (2.28)
Male	P100	1.99 (1.32)	2.05 (1.37)	1.35 (1.41)	1.7 (1.46)	1.84 (1.2)	2.13 (1.3)
	N170	-5.74 (3.05)	-6.54 (3.97)	-5.07 (2.88)	-4.38 (3.37)	-3.11 (3.02)	-3.32 (3.28)

Note：LH：Left hemisphere；RH：Right hemisphere.

1.1 P100 amplitude

The results showed a significant main effect of Stimulus Category, F (2, 136) = 11.867, $p < .001$, $\eta^2_p = .149$ and a significant Stimulus Category * Sex interaction, F (2, 136) = 3.698, $p = .028$, $\eta^2_p = .052$. Further analysis revealed that the P100 amplitude elicited by faces was significantly larger than that elicited by Chinese characters in men, t (35) = 4.4, $p < .001$. Moreover, the P100 amplitude elicited by houses was significantly larger than that elicited by Chinese characters in men, t (35) = 4.245, $p < .001$. Another significant Stimulus Category * Hemisphere interaction, F (2,136) = 3.767, $p = .031$, $\eta^2_p = .052$ was found. Post-hoc t test revealed that only the P100 amplitude elicited by Chinese characters was marginal smaller in the left

hemisphere than in the right, *t* (69) = 1.906, *p* = .061.

1.2 Baseline-to-peak N170 amplitude

The results showed a significant main effect of Stimulus Category, *F*(2,136)=83.213, *p*<.001, η^2_p=.550, with the baseline-to-peak N170 amplitude elicited by faces being larger than that by Chinese characters, *t*(69)=-5.294, *p*<.001, and Houses, *t*(69)=-12.835, *p*<.001. Furthermore, the baseline-to-peak N170 amplitude to Chinese characters was much larger than to Houses, *t*(69)=-7.302, *p*<.001.

A marginally significant Stimulus Category * Hemisphere * Sex interaction, *F*(2,136)=3.197, *p*=.057, η^2_p=.045, a significant Stimulus Category * Sex interaction, *F*(2,136)=5.050, *p*=.008, η^2_p=.069, and a significant Stimulus Category * Hemisphere interaction, *F*(2,136)=6.535, *p*=.004, η^2_p=.088, was also found. The further analysis of Stimulus Category * Hemisphere * Sex interaction in an ANOVA for the factors Hemisphere (Left, Right) and Sex (Women, Men) was conducted separated for face, Chinese character and house study stimuli. For faces as the study stimuli, these analyses demonstrated a significant main effect of gender, *F*(1,68)=5.307, *p*=.024, η^2_p=.072, with a larger responses in men than women. A marginally significant Hemisphere * Sex interaction, *F*(1,68)=3.606, *p*=.062, η^2_p=.050, was also found. Post-hoc *t* tests revealed that the baseline-to-peak N170 amplitude elicited by faces was more right-lateralized in male groups, *t*(35) = 2.091, *p*= .044, but not in female groups, *t*(33) =-.678, *p* = .503. For Chinese characters as study stimuli, a significant main effect of Hemisphere, *F*(1,68)=5.154, *p*=.026, η^2_p=.070, with a larger responses in left hemisphere than that in right hemisphere. Moreover, when the study stimulus was a house, no main effects or interactions were significant.

2 Adaptor stimuli results

The ERP responses elicited by test stimuli (face and Chinese character) were analyzed in an ANOVA for the factors Test Category (Face, Chinese character), Paired Condition [Within category (FF ,CC), Control category (HC, HF)], Hemisphere (Left,

Right) and Sex (Women, Men). The results are shown in Table 4.

Table 4. The p100 and baseline-to-peak N170 amplitude(μV, M（SD）) elicited by the test stimuli

		FF		HF		CC		HC	
		LH	RH	LH	RH	LH	RH	LH	RH
Female	P100	0.86 (1.27)	0.85 (1.42)	1.33 (1.44)	1.49 (1.92)	0.59 (1.15)	1.08 (1.44)	0.91 (1.19)	1.19 (2.0)
	N170	-3.56 (2.46)	-3.48 (2.49)	-5.8 (3.4)	-5.5 (3.86)	-4.51 (2.66)	-3.83 (2.29)	-5.49 (3.29)	-4.73 (3.22)
Male	P100	0.28 (1.75)	0.89 (1.76)	0.47 (1.97)	1.04 (2.46)	-0.07 (1.7)	0.78 (1.98)	0.41 (1.92)	1.07 (2.07)
	N170	-5.53 (3.17)	-5.79 (3.69)	-9.01 (3.29)	-9.55 (4.17)	-6.31 (3.01)	-5.6 (2.83)	-8.49 (3.47)	-7.27 (3.46)

Note：LH：Left hemisphere；RH：Right hemisphere.

2.1 P100 amplitude results of test stimuli

The results yielded a marginally significant main effect of Test category, F (1, 68) = 3.668, p = .060, η^2_p = .051; a significant main effect of Paired condition, F (1, 68) = 14.775, p < .001, η^2_p = .178; and a significant main effect of Hemisphere, F (1, 68) = 6.203, p = .015, η^2_p = .084. A marginally significant Test category * Paired condition * Sex interaction, F (1, 68) = 3.653, p = .061, η^2_p = .051, was found. A repeated-measures ANOVA was conducted for the factors Paired Condition and Sex for face and Chinese character test stimuli, separately. It showed a significant main effect of Paired Condition both for face, F (1, 68) = 9.804, p = .003, η^2_p = .126, and Chinese character stimuli, F (1, 68) = 7.803, p = .007, η^2_p = .103, with the P100 amplitude being smaller when preceded by the within category stimulus than by the control category stimulus.

2.2 Baseline-to-peak N170 amplitude

A repeated measures ANOVA on baseline-to-peak N170 amplitude with factors of Test category, Paired condition, Hemisphere and Sex revealed significant main effects of Paired condition, F(1,68)=166.389, p<.001, η^2_p=.710, and Sex, F(1,68)=14.835,

$p<.001$, $\eta^2_p=.179$.

A significant Test category * Paired condition * Hemisphere * Sex interaction, $F(1,68)=5.194$, $p=.026$, $\eta^2_p=.071$, on the baseline-to-peak N170 amplitude was found. A marginally significant Test category * Hemisphere * Sex interaction, $F(1,68)=3.013$, $p=.087$, $\eta^2_p=.042$, and Test category * Gender interaction, $F(1,68)=3.654$, $p=.060$, $\eta^2_p=.051$, were found. A significant Paired condition * Sex interaction, $F(1,68)=13.753$, $p<.001$, $\eta^2_p=.168$, a significant Test category * Paired condition interaction, $F(1,68)=42.690$, $p<.001$, $\eta^2_p=.386$, and a significant Test category * Hemisphere interaction, $F(1,68)=15.388$, $p<.001$, $\eta^2_p=.185$, were found.

The further analysis of Test category * Paired condition * Hemisphere * Sex interaction in an ANOVA for the factors Paired Condition [Within category (FF, CC), Control category (HC, HF)], Hemisphere (Left, Right) and Sex (Women, Men) was conducted separately for face test stimuli and Chinese character test stimuli. These analyses demonstrated a significant main effect of Sex for both face test stimuli, $F(1,68)=15.947$, $p<.001$, $\eta^2_p=.190$, and Chinese character test stimuli, $F(1,68)=12.169$, $p=.001$, $\eta^2_p=.152$, with the baseline-to-peak N170 amplitude being much larger in men than women. The significant main effect of paired condition was observed for both face test stimuli, $F(1,68)=166.368$, $p<.001$, $\eta^2_p=.71$, and Chinese character test stimuli, $F(1,68)=66.942$, $p<.001$, $\eta^2_p=.496$. The significant Paired condition * Sex interaction, was observed for both face test stimuli, $F(1,68)=11.172$, $p=.001$, $\eta 2_p=.141$, and Chinese character test stimuli, $F(1,68)=7.949$, $p=.006$, $\eta^2_p=.105$. Post-hoc *t* tests revealed that baseline-to-peak N170 amplitude in men are much larger than that in women in both paired condition for face test stimuli(FF, $t(68)=3.188$, $p=.002$; HF, $t(68)=4.395$, $p<.001$), and Chinese character test stimuli(CC, $t(68)=3.013$, $p=.004$; HC, $t(68)=3.694$, $p<.001$). Moreover, the baseline-to-peak N170 amplitude elicited by test stimuli was much smaller when preceded by within category stimulus than preceded by control category stimulus in both groups for face test stimuli (Women：$t(33)=8.310$， $p<.001$;　Men: $t(35)=10.077$, $p<.001$), and for Chinese character test stimuli(Women：$t(33)=3.472$，$p=.001$; Men: $t(35)=8.563$, $p<.001$). For Chinese character as test stimuli, the ANOVA analysis also revealed a significant main effect of Hemisphere,

$F(1,68)=9.843$, $p=.003$, $\eta 2_p=.126$, and a marginally significant Paired condition * Hemisphere interaction, $F(1,68)=3.81$, $p=.055$, $\eta 2_p=.053$. Post-hoc *t* tests revealed that baseline-to-peak N170 amplitude elicited by Chinese character test stimuli in both Paired Conditions was much larger in left hemisphere than that in right hemisphere(CC, $t(69)=-2.644$, $p=.010$; HC, $t(69)=-3.424$, $p<.001$). Moreover, in both hemispheres, for Chinese character as test stimuli, the baseline-to-peak N170 amplitude was much larger when preceded by house than by Chinese character (LH: $t(69)=8.211$, $p<.001$;RH: $t(69)=6.389$, $p<.001$).
